# Nine golden codes: improving the accuracy of Helicopter Emergency Medical Services (HEMS) dispatch—a retrospective, multi-organisational study in the East of England

**DOI:** 10.1186/s13049-023-01094-w

**Published:** 2023-06-12

**Authors:** Christopher T. Edmunds, Kate Lachowycz, Sarah McLachlan, Andrew Downes, Andrew Smith, Rob Major, Edward B. G. Barnard

**Affiliations:** 1 Department of Research, Audit, Innovation, and Development, East Anglian Air Ambulance, Gambling Close, Norwich Airport, Norwich, NR6 6EG UK; 2 Essex & Herts Air Ambulance Trust, Colchester, Essex, UK; 3 Anglia Ruskin University, Cambridge, UK; 4 University of East Anglia, Norwich, UK; 5 Magpas Air Ambulance, Huntingdon, UK; 6 Academic Department of Military Emergency Medicine, Royal Centre for Defence Medicine (Research & Clinical Innovation), Birmingham, UK; 7 Emergency Department, Cambridge University Hospitals NHS Foundation Trust, Cambridge, UK

**Keywords:** Air ambulances, Emergency medical dispatch, Emergency medical services, Prehospital emergency care

## Abstract

**Background:**

Helicopter Emergency Medical Services (HEMS) are a limited and expensive resource, and should be intelligently tasked. HEMS dispatch was identified as a key research priority in 2011, with a call to identify a ‘general set of criteria with the highest discriminating potential’. However, there have been no published data analyses in the past decade that specifically address this priority, and this priority has been reaffirmed in 2023. The objective of this study was to define the dispatch criteria available at the time of the initial emergency call with the greatest HEMS utility using a large, regional, multi-organizational dataset in the UK.

**Methods:**

This retrospective observational study utilized dispatch data from a regional emergency medical service (EMS) and three HEMS organisations in the East of England, 2016–2019. In a logistic regression model, Advanced Medical Priority Dispatch System (AMPDS) codes with ≥ 50 HEMS dispatches in the study period were compared with the remainder to identify codes with high-levels of HEMS patient contact and HEMS-level intervention/drug/diagnostic (HLIDD). The primary outcome was to identify AMPDS codes with a > 10% HEMS dispatch rate of all EMS taskings that would result in 10–20 high-utility HEMS dispatches per 24-h period in the East of England. Data were analysed in R, and are reported as number (percentage); significance was *p* < 0.05.

**Results:**

There were* n* = 25,491 HEMS dispatches (6400 per year), of which *n* = 23,030 (90.3%) had an associated AMPDS code. *n* = 13,778 (59.8%) of HEMS dispatches resulted in patient contact, and *n* = 8437 (36.6%) had an HLIDD. 43 AMPDS codes had significantly greater rates of patient contact and/or HLIDD compared to the reference group. In an exploratory analysis, a cut-off of ≥ 70% patient contact rate and/or ≥ 70% HLIDD (with a > 10% HEMS dispatch of all EMS taskings) resulted in 17 taskings per 24-h period. This definition derived nine AMPDS codes with high HEMS utility.

**Conclusion:**

We have identified nine ‘golden’ AMPDS codes, available at the time of initial emergency call, that are associated with high-levels of whole-system and HEMS utility in the East of England. We propose that UK EMS should consider immediate HEMS dispatch to these codes.

**Supplementary Information:**

The online version contains supplementary material available at 10.1186/s13049-023-01094-w.

## Background

Physician-staffed prehospital teams have existed for over 50 years. In 1967, there were 20,000 motor vehicle collision deaths in Germany, leading to emergency doctors demanding earlier and better treatment of injured patients. In response, the first Helicopter Emergency Medical Service (HEMS) ‘Christoph 1’ became operational in Munich in 1970 [1]. Since then, over 2.2 million HEMS missions have taken place in Germany, and similar physician-staffed HEMS have been developed, predominantly in Europe and Australasia.

The primary rationale for HEMS is the rapid deployment of a specialist team over a large geographic area, with the assumption that physicians working alongside paramedics infer better patient outcomes compared to paramedics alone. The latter point is multi-factorial, and includes higher-level decision-making [2, 3], and interventions that are either physician-level specific or require drugs that legally require their presence [3–5]; an example in the UK is prehospital emergency anaesthesia (PHEA) [6, 7]. There is contradictory evidence that physician-staffed teams are associated with better patient outcomes following trauma [8–14], and out-of-hospital cardiac arrest [15–18]. However, the heterogeneity of systems makes conclusive synthesis of these data inappropriate, and physician-staffed HEMS remain a key component of many emergency medical systems (EMS).

HEMS are a limited and expensive resource [19–21]. It is therefore important to ensure that these teams are intelligently dispatched [22], which may in time also lead to a better understanding of which patients have the greatest likelihood of benefit—these concepts are somewhat inter-dependent. HEMS dispatch was identified as a key research priority in 2011, with a call to identify a ‘general set of criteria with the highest discriminating potential’ [23]. It has again been identified as a key research priority in 2023 suggesting limited progress in this area over the last decade [24]. Since 2011, London’s Air Ambulance has reported that call-interrogation and crew request dispatch models were more accurate for HEMS utilization compared to an immediate dispatch model based on mechanism of injury. However, this was at the expense of delaying dispatch [25], which is likely to dilute HEMS benefit. There is also UK data that suggests non-clinical dispatchers may improve HEMS dispatch accuracy when using a bespoke algorithm [21]. However, clinicians in dispatch systems often undertake additional roles; for example, remote clinical advice to EMS. Overall, there is a paucity of data that could be used to increase the accuracy of HEMS dispatch [26, 27], possibly owing to the intrinsic complexity of these systems.

Therefore, the challenge lies in increasing the accuracy of HEMS-appropriate taskings using information available from the initial emergency call. The positives of which may include: better patient outcomes (by reducing under-triage), greater availability of HEMS for those with the highest chance of benefit (by reducing over-triage) [28], reducing costs associated with helicopter dispatch [29], and reducing unnecessary emergency aviation risk to medical teams [30]. The objective of this study was to define the dispatch criteria available at the time of the initial emergency call with the greatest HEMS utility using a large, regional, multi-organisational dataset in the UK.

## Methods

### Emergency medical system

The East of England is a 20,000 km^2^ geographic area, containing a population of 6.3 million people (June 2021) [31]. The statutory regional EMS is the East of England Ambulance Service NHS Trust (EEAST), which receives approximately 4000 calls per day, and has been previously described [18]. EEAST is supported by three HEMS charities operating from five operational bases: East Anglian Air Ambulance (EAAA) [32], Magpas Air Ambulance (Magpas) [33], and Essex & Herts Air Ambulance (EHAAT) [34]. The core of each team consists of a prehospital-trained physician and a critical care paramedic (CCP) with a minimum of three years’ postgraduate experience and role-specific training [6]. Occasionally, HEMS resources may be staffed by one or two CCPs without a physician. During the study period, HEMS teams typically responded by helicopter during the day and rapid response vehicle (RRV) at night, during periods of aircraft unavailability, and when an RRV dispatch was thought to be advantageous.

### Dispatch system

EEAST uses the Advanced Medical Priority Dispatch System (AMPDS, Priority Dispatch Corporation, Salt Lake City, Utah, USA, version 13.3). AMPDS provides a scripted caller interrogation protocol to prioritise calls and allocate EMS resources to incidents via the linked ProQA (Priority Dispatch Corp., ProQAv 5.1.1.44) software system. The data end-point is a ‘number-letter-number’ sequence (AMPDS code) that describes the category, severity (in reference to the speed and configuration of EMS response required, A to E), and sub-type of the incident. For example, a cardiac or respiratory arrest (patient not breathing) would be coded as ‘09-E-01’; ‘09’ is the AMPDS code category of ‘cardiac or respiratory arrest’, ‘E’ is a capability of ‘Advanced Life Support and special units’ with a response of ‘hot (multiple units) plus other first responders’, and the ‘01’ sub-type of this code refers to ‘not breathing at all’.

AMPDS codes are available either during or directly after the initial emergency call, and therefore present an appropriate target in modelling HEMS dispatch utility using immediately available information. These codes are typically set within the initial call handling process, but they can be manually changed if more information becomes available during the triage process.

HEMS are dispatched at the discretion of the EEAST Critical Care Desk (CCD), which is routinely staffed by a HEMS-dispatcher and a clinically-active CCP. However, at times CCD may be staffed by a dispatcher without a CCP. CCD personnel continuously review EEAST emergency calls 24 h per day, with the aim of identifying incidents believed to be most appropriate for HEMS, either as an ‘immediate’ dispatch based on AMDPS codes and other information in the Computer Aided Dispatch (CAD) system, or as an ‘interrogate’ (additional information from the caller), as well as responding to EMS requests for a HEMS co-response (crew request).

### Data inclusion criteria

All HEMS dispatches by EEAST (EAAA, Magpas, EHAAT) during a four-year period (2016–2019) where an AMPDS code was available. In order to ensure that AMPDS codes with the highest system utility were identified, EEAST provided complete system (EMS and HEMS) AMPDS code dispatches for a 12-month period (2021)—the ‘EMS reference group’.

### Data collection

All services use HEMSbase (MedicOne Systems Ltd, UK) electronic medical record software. The following data were extracted from HEMSbase. HEMS taskings: incident type, call result (stand down, or patient attended), callsign (helicopter, or RRV), job timings (day—0700–1900, or night—1900–0700), CCD team composition (CCP team, or dispatcher alone), and the EEAST unique case identification number (CAD date/number). The following data were additionally collected for all patients attended by HEMS: patient age and gender, all drugs given, all interventions performed, and attendance result (air conveyance, ground escort, scene assist (HEMS team did not accompany patient to hospital), and stood down (before mobile, en route or at scene). The CAD number/dates were shared with EEAST in order to obtain the AMPDS codes. Combined data from the three HEMS were collated into Excel data sheets and stored on a secure server protected by double-authenticator security and only accessible to the team working on the project; all data were anonymised.

### Definitions

HEMS-level intervention/drug/diagnostic (HLIDD)—all HEMS-delivered actions were compared against Schedule 17 of the UK Human Medicines Regulations 2012 (exemption for registered paramedics from the restrictions on administration of prescription-only medicines) [35, 36], and the Joint Royal Colleges Ambulance Liaison Committee (JRCALC) clinical guidelines October 2022 [37], to identify a list of HLIDD; additionally, we included helicopter conveyance to hospital. A list of HLIDDs is in Additional file 1. Not all HLIDDs are routinely delivered by every HEMS in this region.

Patient contact was defined as clinical contact with the patient by a member of the HEMS team. Stand-down was defined as no clinical contact (either stood-down before mobile, en route, or at scene). AMPDS codes were defined as the complete three-part data (09-E-01). AMPDS categories were defined as the first data descriptor of the AMPDS code (09). High system utility was pre-defined as AMPDS codes that had > 10% HEMS dispatch rate of all EMS taskings in the EMS reference group (2021), in order to ensure that codes identified in the primary outcome had significant whole-system frequency; for example, code 09E01 was found to comprise of 13.7% of all HEMS tasking by EEAST.

Owing to the lack of previously published data in the area of HEMS dispatch we planned to undertake an exploratory analysis for the primary outcome of paired ‘patient contact rate’ and ‘HLIDD’ proportions (60%, 70%, 80%, 90%) to identify codes that would result in an optimal number of high-utility HEMS dispatches in a 24-h period. This was pre-defined as 10–20 HEMS dispatches (two to four dispatches on average for each of the five HEMS teams in the East of England). The authors derived optimal dispatch frequency through consensus based on operational experience, which also takes into account the potential limitations to activity (weather, fuel, equipment and drug restocking during a duty period).

### Primary outcome

The primary outcome was to identify AMPDS codes with a > 10% HEMS dispatch rate of all EMS taskings that would result in 10–20 high-utility HEMS dispatches per 24-h period in the East of England.

### Secondary outcomes

The secondary outcomes were to identify plausible associations with the chance of patient contact and the chance of an HLIDD using individual logistic regression models for the ten most frequently HEMS-dispatched AMPDS categories. The pre-specified variables of interest were: age, sex, time of day (day, night), HEMS transport platform (helicopter, RRV), and CCD team composition (dispatcher and CCP, lone dispatcher).

### Data analysis

For the primary outcome, the EMS reference group data were interrogated to identify AMPDS codes with a > 10% HEMS dispatch rate in 2021. In the HEMS dispatch data (2016–2019), codes were identified that resulted in ≥ 50 HEMS dispatches in the four-year period. Codes with < 50 HEMS dispatches were used as the reference group in two logistic regression models. The first model identified codes with a significantly high rate of patient contact compared with the reference group (using a binary outcome of 1 = patient seen, 0 = stand down). A second logistic regression analysis identified codes with a significantly high rates of HLIDD compared with the reference group (using a binary outcome of 1 = HLIDD, 0 = no HLIDD) for jobs where a patient was seen. We then undertook an exploratory analysis to determine the optimal patient contact and/or HLIDD proportions (with a > 10% HEMS dispatch rate of all EMS taskings) that would result in 10–20 high-utility HEMS dispatches in 24 h.

For the secondary outcomes, all variables in the HEMS dispatch data (2016–2019) were included in further individual logistic regression models for each of the ten most prevalent AMPDS categories with two outcomes—patient contact and HLIDD. Age was separated into bins (< 16, 16–55, > 55 years old) based on clinically pragmatic ranges (all other variables were binary). Taskings that resulted in more than one patient being treated were excluded from the analysis of age and sex. A logistic regression model was built for each of the ten AMPDS categories, starting with all variables followed by sequential elimination, to retain only those with significance for the outcome (patient contact and/or HLIDD). For all models, the assumptions of logistic regression were tested, checking for linear relationships in the logit of the outcomes, unduly influential values and multicollinearity. Plausible interactions were tested with likelihood ratio tests to determine the final best model. Missing data were handled as ‘missing at random’. Significance was predefined as *p* < 0.05 throughout.

Data have been reported as number (percentage), and median [interquartile-range] as appropriate. Results of logistic regression have been reported as an adjusted odds ratios (OR) with 95% confidence intervals (95%CI), both derived from coefficients in the logistic regression model. Data manipulation and statistical analyses were performed using the R statistical programming language (R Core Team [2018]; R: A language and environment for statistical computing [R Foundation for Statistical Computing, Vienna, Austria]).

## Results

**Fig. 1:**
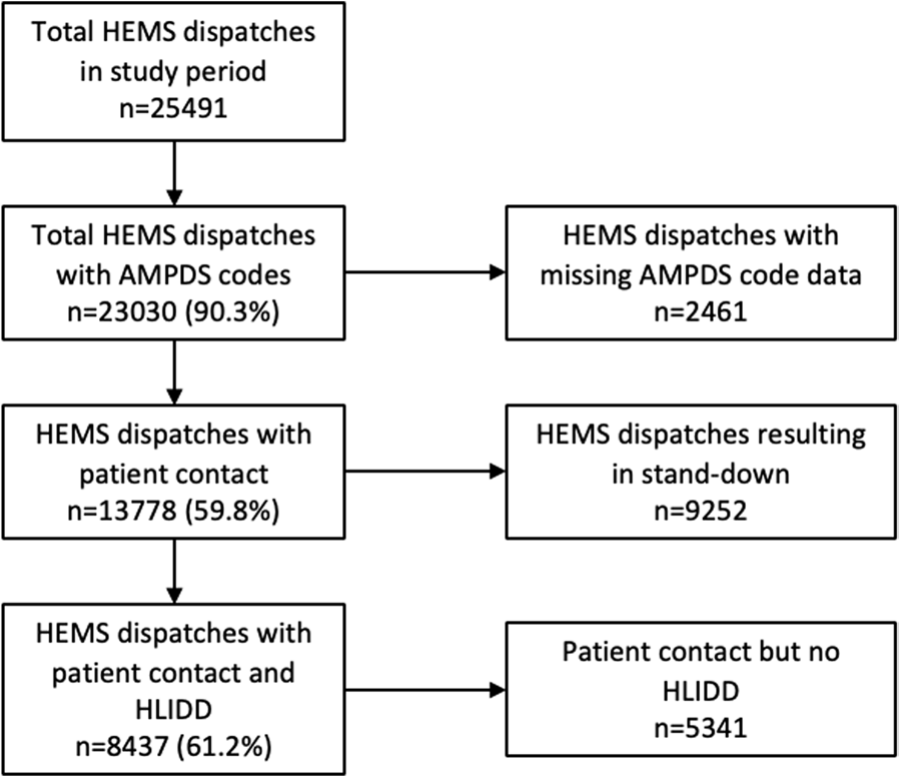
HEMS dispatches in the East of England (2016–2019) with complete AMPDS code data. HEMS patient contact, and HEMS-level intervention/drug/diagnostic (HLIDD). *HEMS* Helicopter Emergency Medical Service. *AMPDS* Advanced Medical Priority Dispatch System. Stand-down—the HEMS team were not required and were cancelled before patient contact

During the four-year study period there were *n* = 25,491 HEMS dispatches (6400 per year), of which *n* = 20,030 (90.3%) had an associated AMPDS code, Fig. 1.

### HEMS dispatches

Approximately half of all HEMS dispatches were immediate, and the majority occurred when the dispatch team included a CCP. More dispatches occurred during the day shift (0700–1900) compared to the night shift, and the HEMS transport platform was a helicopter in more than half of cases. The 23,030 HEMS dispatches included *n* = 678 unique AMPDS codes, and the most prevalent AMPDS categories were cardiac/respiratory, road traffic collisions (RTC), and falls, Table 1.

**Table 1:** A description of HEMS dispatches in the East of England 2016–2019, *n* = 23,030

	n (%)
**Dispatch type**	
Immediate	12,463 (54.1)
Interrogate	5428 (23.6)
Crew request	4918 (21.4)
**Dispatch characteristics**	
CCP in dispatch team	20,912 (90.8)
Day shift (0700-1900)	16,552 (71.7)
Helicopter	14,750 (64.1)
RRV	8280 (36.0)
**AMPDS category (category number)**	
Cardiac/respiratory (09)	5299 (23.0)
RTC (29)	4951 (21.5)
Falls (17)	2491 (10.8)
Stab/gunshot (27)	1532 (6.7)
Unconscious/fainting (31)	1204 (5.2)
Convulsions/fitting (12)	1183 (5.1)
Traumatic injuries (30)	1114 (4.8)
Breathing problems (06)	902 (3.9)
Burns (07)	361 (1.6)
Chest pain (10)	347 (1.5)
Other (> 10 in rank order)	3646 (15.8)

*CCP* Critical Care Paramedic, *RRV* rapid response vehicle (car), *AMPDS* Advanced Medical Priority Dispatch System, *RTC* road traffic collision

### HEMS dispatches with patient contact

*N* = 13,778 (59.8%) of HEMS dispatches resulted in patient contact, and therefore the stand-down rate was approximately 40%. The median age was 49 [29–65] years and included *n* = 1060 (7.7%) patients under 16 years old (*n* = 63 missing age data). There were *n* = 9109 (66.1%) male patients and five (0.04%) were recorded as transgender (*n* = 86 missing gender data). *n* = 8437 (61.2%) of missions with patient contact resulted in use of HLIDD for at least one patient, and a total of *n* = 8967 patients received an HLIDD—this number is greater than the ‘missions with patient contact that resulted in an HLIDD’ number as some missions included treatment of more than one patient, Table 2.

**Table 2:** Patients attended by HEMS in the East of England (2016–2019) who received a HEMS-level intervention/drug/diagnostic (HLIDD), *n* = 8967. Only including HLIDD received by > 400 patients

HLIDD	N	%
Endotracheal tube	3883	43.3
Advanced analgesia	2576	28.7
Conveyed by helicopter	2463	27.5
PHEA	2194	24.5
POCUS (Cardiac)	1070	11.9
Gastric Tube	1047	11.7
Procedural sedation	867	9.7
Oesophageal Temperature Probe	842	9.4
Intravenous antimicrobial	771	8.6
Thoracostomy	651	7.3
Intravenous electrolyte	643	7.2
Splintage	558	6.2
POCUS (FAST)	513	5.7
POCUS (Lung)	492	5.5
Radial arterial line	471	5.3

*HLIDD* HEMS-level intervention/drug/diagnostic (complete list at Additional file 1); advanced analgesia includes intravenous fentanyl and inhaled methoxyflurane (Penthrox); *PHEA* prehospital emergency anaesthesia (drug-assisted intubation); *POCUS* point-of-care ultrasound; gastric tube includes oro and nasogastric decompression tubes; intravenous electrolyte includes magnesium, calcium chloride, and sodium bicarbonate; intravenous antimicrobial includes coamoxiclav, ceftriaxone, and acyclovir

### Primary outcome

A small proportion, *n* = 84 (12.4%), of AMPDS codes resulted in ≥ 50 HEMS dispatches per code, but represented more than three-quarters of all HEMS dispatches (*n* = 18,240, 79.2%) as total number.

43 AMPDS codes were identified that had significantly greater rates of patient contact and/or HLIDD compared to the reference group. These codes were associated with 11,640 (50.5%) HEMS dispatches in the study period. In the exploratory analysis, a cut-off of ≥ 70% patient contact rate and/or ≥ 70% HLIDD (with a > 10% HEMS dispatch of all EMS taskings) resulted in 17 taskings per 24-h period. Additional file 2 includes the sensitivity analysis to explore the impact of thresholds on estimated tasking numbers per 24-h period. This definition derived nine ‘golden’ AMPDS codes with a high whole-system and HEMS utility, and an optimal frequency of HEMS dispatches, Table 3.

**Table 3:** AMPDS codes with a significantly greater chance of HEMS patient contact and/or HEMS-level intervention/drug/diagnostic (HLIDD) compared to the reference group (2016–2019). Nine ‘golden’ codes had a ≥ 70% rate of patient contact and/or HLIDD (2016–2019) and were > 10% of EEAST incidents

AMPDS code category	AMPDS code
Burns	07-C-03
Cardiac/respiratory	09-E-01
Convulsions/fitting	12-D-01
Falls	17-D-02P, 17-D-06, 17-D-06P
RTC	29-D-06, 29-D-06 V, 29-D-07 V

*AMPDS* Advanced Medical Priority Dispatch System, *HEMS* helicopter emergency medical service, *EEAST* East of England Ambulance Service NHS Trust, *RTC* road traffic collision

A complete description of the 43 AMPDS codes with significantly high rates of patient contact or HLIDD is in Additional file 3.

### Secondary outcomes

The ten most prevalent AMPDS categories of HEMS dispatch were analysed, *n* = 19,384 (84.2%) of taskings. Only variables that had significance in the univariate models for each AMPDS category (not presented) were included in the multivariate analyses.

HEMS at night (compared to day) and dispatch by RRV (compared to helicopter) both had a significantly lower chance of patient contact and HLIDD for several AMPDS categories. The exception to this was road traffic collisions, which had significantly higher rates of patient contact at night compared day.

The presence of a CCP in the CCD team had no effect on the chance of patient contact or HLIDD, other than for penetrating injury codes (stab/gunshot, 27-X-XX) for which a CCP on the desk was associated with an increased chance of patient contact but had no effect on the chance of HLIDD.

Age < 16 years old (compared to 16–55 years old) was associated with a lower chance of HLIDD for the majority of categories, and age > 55 years old had a greater chance of HLIDD for cardiac/respiratory and traumatic injury categories, and lower for burns. Male sex (compared with female sex) was associated with a greater chance of HLIDD for cardiac/respiratory, road traffic collisions and convulsions/fitting categories, Tables 4 and 5.

**Table 4:** Variables associated with significant high or low rates of HEMS patient contact (multivariate model)

AMPDS category/n	Night (ref: day)	RRV (ref: helicopter)	Dispatch desk CCP (ref: no CCP)
	Adjusted odds ratio (95% confidence interval)		
06–Breathing problems *n* = 902	0.6 (0.4–0.8)	ns	ns
07—Burns *n* = 361	0.5 (0.3–0.9)	0.5 (0.3–0.8)	ns
09—Cardiac/respiratory *n* = 5299	0.8 (0.7–0.9)	0.8 (0.7–0.9)	ns
10–Chest pain *n* = 347	0.3 (0.1–0.4)	ns	ns
12—Convulsions/fitting *n* = 1183	ns	ns	ns
17—Falls *n* = 2491	0.5 (0.4–0.7)	0.8 (0.6–0.9)	ns
27—Stab/gunshot *n* = 1532	0.7 (0.6–0.9)	ns	**1.6 (1.1**–**2.5)**
29—RTC *n* = 4951	0.8 (0.7–0.9)	0.8 (0.7–0.9)	ns
30—Traumatic injuries *n* = 1114	ns	0.5 (0.4–0.7)	ns
31—Unconscious/fainting *n* = 1204	0.6 (0.5–0.8)	ns	ns

*HEMS* Helicopter Emergency Medical Service, *AMPDS* Advanced Medical Priority Dispatch System, *RRV* rapid response vehicle, *CCP* critical care paramedic, *ns* not significant (*p*-value ≥ 0.05). An odds ratio < 1 is a reduced chance of the outcome (not highlighted bold); an odds ratio > 1 is an increased chance of the outcome (highlighted bold)

**Table 5:** Variables associated with significant high or low rates of HEMS-level intervention/drug/diagnostic (multivariate model)

AMPDS category / n	Night (ref: day)	RRV (ref: helicopter)	Dispatch desk CCP (ref: no CCP)	Male (ref: female)	Age < 16 years (ref: 16–55 years)	Age > 55 years (ref: 16–55 years)
	Adjusted odds ratio (95% confidence interval)					
06—Breathing problems *n* = 493	ns	0.4 (0.3–0.6)	ns	ns	0.1 (0.1–0.3)	ns
07—Burns *n* = 200	0.3 (0.1–0.6)	ns	ns	ns	0.5 (0.2–0.9)	0.4 (0.2–0.9)
09—Cardiac/respiratory *n* = 3128	ns	0.7 (0.6–0.8)	ns	**1.2 (1.1**–**1.4)**	0.6 (0.5–0.9)	**1.3 (1.1**–**1.5)**
10—Chest pain *n* = 277	ns	0.3 (0.2–0.6)	ns	ns	ns	ns
12—Convulsions/fitting *n* = 760	ns	ns	ns	**1.4 (1.1**–**1.9)**	0.5 (0.3–0.7)	ns
17—Falls *n* = 1785	0.7 (0.6–0.9)	0.6 (0.5–0.8)	ns	ns	0.4 (0.3–0.6)	ns
27—Stab/gunshot *n* = 549	ns	0.3 (0.2–0.4)	ns	ns	ns	ns
29—RTC *n* = 3018	**1.3 (1.0**–**1.5)**	0.5 (0.4–0.6)	ns	**1.5 (1.2**–**1.8)**	ns	ns
30—Traumatic injuries *n* = 786	0.5 (0.3–0.9)	0.6 (0.3–0.9)	ns	ns	0.6 (0.4–0.9)	**1.4 (1.1**–**2.1)**
31—Unconscious/fainting *n* = 682	ns	0.7 (0.5–0.9)	ns	ns	0.1 (0.1–0.3)	ns

*HEMS* Helicopter Emergency Medical Service, *AMPDS* Advanced Medical Priority Dispatch System, *RRV* rapid response vehicle, *CCP* critical care paramedic, *ns* not significant (*p*-value ≥ 0.05). An odds ratio < 1 is a reduced chance of the outcome (not highlighted bold); an odds ratio > 1 is an increased chance of the outcome (highlighted bold)

## Discussion

We have used large, multi-organisational, data to identify nine ‘golden’ AMPDS codes with significant utility in improving the accuracy of HEMS dispatch with respect to patient contact rates and HEMS-level intervention/drug/diagnostic rates. In addition, we have demonstrated some important associations with these outcomes for age, sex, time of day, HEMS transport type, AMPDS categories, and the presence of a clinician (a critical care paramedic) in the dispatch team.

There is a distinct paucity of published data in the area of HEMS dispatch despite this being an expensive and limited resource where research has been identified as a priority for the last decade. It is likely that this is due to a combination of factors, which includes the volume, complexity, and availability of data in these systems. We have therefore had to make pragmatic consensus decisions on appropriate data definitions, together with an exploratory analysis of HEMS dispatches per 24-h time period to determine the optimal cut-off for specificity of the primary outcome, which has a ≤ 30% over-triage rate. By happenstance, this over-triage rate is compliant with the American College of Surgeon’s Committee on Trauma (ACS CoT) guideline for the triage of prehospital major trauma (25–35% over-triage) [38]. However, as we were only able to determine the patient contact and HLIDD rates for patients that HEMS were dispatched to we are unable to determine the under-triage rate (i.e. what proportion of EMS dispatches should have had a HEMS patient contact and/or HLIDD, but did not). Arguably, this is a more important metric when considering the performance of a triage system, and the ACS CoT recommends an under-triage rate of < 5% [38]. It is very likely that under-triage in UK EMS for HEMS patient contact and HLIDD is much higher than 5%, but there are no data that verify this assumption. However, we have been able to identify an easily applied method to reduce the over-triage rate from > 40% (HEMS stand-down rate in our complete data) to ≤ 30%, whilst also assisting the very early identification of emergency calls with a greater chance of leading to HEMS-level actions. We believe that these data are unique in addressing the paucity of HEMS dispatch data [26], and the associated 2011 and 2023 prehospital research priority [23].

The main controversy in this area of research is whether there is patient-centered benefit from HEMS compared to EMS-only attendance. Whilst the existing evidence is perhaps equivocal [8–18], a key factor in robustly answering this question (and in providing optimal patient care across a system) is ensuring that HEMS are dispatched to the right patients (i.e., those who are most likely to benefit), which may be aetiology-dependent [12, 15]. Exact aetiology is often not available at the time of the initial emergency call, which therefore necessitates using a time-sensitive surrogate, which in the case of this study was AMPDS codes. Overall, there is a low level of evidence supporting the accuracy of dispatch systems in identifying acuity [27]. However, we have been able to rely on the pragmatic judgement of prehospital clinicians in the need for HEMS patient contact and HEMS interventions, the frequency of which we have been able to use to identify an optimal number (for operational delivery) of AMPDS codes associated with the highest levels of HEMS utility. We cannot control for the major constraint of not knowing the under-triage rate for these codes, and they should be prospectively tested in multiple settings to confirm their utility. The expectation is that identification of high-utility AMPDS codes is a step towards a better understanding of the benefit of HEMS.

We observed that night and RRV taskings, compared to day and helicopter respectively, are both associated with lower patient contact and HLIDD rates overall. This effect has been previously described in the UK and Norway [19, 39], in contrast to The Netherlands where no day-night effect has been reported [28]. The lower rates for RRV taskings, even after adjusting for time-of-day remain unexplained, but it may be that standing-down a road vehicle response is perceived to be more acceptable that re-routing a helicopter. Our data also suggests that the absence of a clinician in dispatch has no effect on the chance of HEMS patient contact for most taskings, with the notable exception of the stab/gunshot call category. Furthermore, there was no signal across any of the ten most prevalent AMPDS categories that this variable had any effect on the chance of a HLIDD (including stab/gunshot). This somewhat agrees with previous UK HEMS data [21], but as there was a CCP in dispatch for > 90% of HEMS taskings, this may represent a type-2 error (small numbers), and we are also unable to control for the likelihood that non-clinical dispatchers have learned from co-dispatching with a CCP for the vast majority of duties. Therefore, we do not believe that our data support non-clinician HEMS dispatch.

We observed significantly higher rates of HLIDD in patients > 55 years old in two AMPDS categories: cardiac/respiratory, traumatic injuries. The first of these is likely to represent a higher acuity of age-related aetiology in these categories, and the latter is likely to reflect the Silver Trauma phenomenon [40]. Older age has previously been identified in UK HEMS as a potentially useful dispatch criteria [41], and our data provide support for this in a more specific and actionable way.

In the East of England, we have used the nine ‘golden’ codes to better inform clinicians on the Critical Care Desk in their decisions around HEMS dispatch. It is anticipated that this will reduce the over-triage rate by approximately 10% (as per our retrospective findings). It may also be useful to utilize these codes as a framework for key performance indicators in HEMS dispatch.

The major limitation of this study is that we were only able to interrogate data for taskings that HEMS had been dispatched on. Therefore, it is possible that patients not attended by HEMS in the study period would have had AMPDS codes assigned that would meet the utility criteria. However, the large data capture over four years for three HEMS organsiations operating five teams somewhat mitigates this. Some AMPDS codes that are used very infrequently, and were therefore excluded from this analysis, are likely to be important when considering HEMS dispatch. Examples include codes relating to major or mass casualty incidents, and there are others (for example: ‘34—automatic crash notification’) that may become more important in the future as technology develops [42]. Our data are likely to have generalizability to HEMS systems that operate a physician-paramedic model in mixed urban and rural geographies that use AMPDS for dispatch. However, these data have less utility in non-AMPDS dispatch systems (for example, those using NHS Pathways in some regions of the UK) without robust mapping of AMPDS and non-AMPDS codes. In addition, whilst the East of England includes a broad mix of urban and rural populations, it is very flat. Therefore, our data may have reduced generalizability in more mountainous geographies where the limitations of patient accessibility, weather, and potentially a different epidemiology of disease and trauma may have a different impact on important dispatch codes.

We have identified nine ‘golden’ AMPDS codes, available at the time of initial emergency call, that are associated with high-levels of whole-system and HEMS utility in the East of England. We propose that UK EMS should consider immediate HEMS dispatch to these codes, or automated notification to HEMS dispatchers when these codes are generated from emergency calls within a robust governance framework.

## Supplementary Information

**Additional file 1**. HEMS-level intervention/drug/diagnostic. A list of all HLIDDs used within this study.

**Additional file 2**. Sensitivity analysis to explore impact of thresholds on estimated tasking numbers/24 h. Demonstration of the impact of specific thresholds for intervention and/or patient contact on code availability.

**Additional file 3**. AMPDS codes with significantly high rates of patient contact and/or HLIDD. A table listing the p value for both HLIDD and patient contact for specific codes.

## Data Availability

Data are available on reasonable request.

## Abbreviations

HEMS: Helicopter Emergency Medical Services
EMS: Emergency medical service
AMPDS: Advanced Medical Priority Dispatch System
HLIDD: HEMS-level intervention/drug/diagnostic
PHEA: Prehospital emergency anaesthesia
EEAST: East of England Ambulance Service NHS Trust
EAAA: East Anglian Air Ambulance
Magpas: Magpas Air Ambulance
EHAAT: Essex & Herts Air Ambulance
CCP: Critical Care Paramedic
RRV: Rapid Response Vehicle
RTC: Road traffic collisions
POCUS: Point of Care Ultrasound
ACS CoT: American College of Surgeon’s Committee on Trauma
