# Supplementary material for: Nine golden codes: improving the accuracy of Helicopter Emergency Medical Services (HEMS) dispatch—a retrospective, multi-organisational study in the East of England
Source: Scand J Trauma Resusc Emerg Med. 2023 Jun 12;31:27. doi: 10.1186/s13049-023-01094-w (PMC10258975; doi:10.1186/s13049-023-01094-w)
Supplement: Supplementary file 1 — Additional file 1. HEMS-level intervention/drug/diagnostic. A list of all HLIDDs used within this study. [file 13049_2023_1094_MOESM1_ESM.pdf]

Additional File 1: HEMS-level intervention/drug/diagnostic (HLIDD)

Interventions

Access	Femoral, IJV, Arterial Line
Airway	Surgical Airway, Needle Cricothyroidotomy, Apnoeic oxygenation, Aerogen, Endotracheal tube, Rapid Sequence Intubation
Defibrillation	Pacing
Gastric tube	Nasogastric. Orogastric
Haemorrhage Control	Packing Max-fax, Suturing
Monitoring	Temperature probe – Rectal/Oesphageal
Nerve Block	Axillary, Femoral, Finger
Sedation	
Splintage	Benecast – Arm/Leg
Surgical	Amputation, Resuscitative Thoracotomy, Escharotomy, Intercostal Drain, Casearean Section
Thoracostomy	
Transport	Carried by air
Ultrasound	Cardiac, Vascular Access, Lung, FAST, Aorta, SPEAR

Drugs

Aciclovir, Adenosine, Beriplex, Blood PRC, Calcium Chloride, Ceftriaxone, Co-Amoxiclav, Cyclizine, Dexamethasone, Ephedrine, Fentanyl, Haloperidol, Heparin, Ketamine, Labetolol, Levetiracetam 500mg/5ml, Lidocaine 1%, Magnesium, Metaraminol, Metoprolol, Midazolam, NaCl 5%, Penthrox, Phenytoin Sodium, Plasma, Propofol, Rocuronium, Salbutomol IV, Sodium Bicarbonate, Suxamethonium, Thiopentone
