# Supplementary material for: Nine golden codes: improving the accuracy of Helicopter Emergency Medical Services (HEMS) dispatch—a retrospective, multi-organisational study in the East of England
Source: Scand J Trauma Resusc Emerg Med. 2023 Jun 12;31:27. doi: 10.1186/s13049-023-01094-w (PMC10258975; doi:10.1186/s13049-023-01094-w)
Supplement: Supplementary file 2 — Additional file 2. Sensitivity analysis to explore impact of thresholds on estimated tasking numbers/24 h. Demonstration of the impact of specific thresholds for intervention and/or patient contact on code availability. [file 13049_2023_1094_MOESM2_ESM.pdf]

Additional File 2: Sensitivity analysis to explore impact of thresholds on estimated tasking numbers/24 hrs

**All statistically significant codes. No thresholds = 340 incidents/24hrs**<br><br>**43 codes**<br><br>06D02, 07C03, 09D01, 09E01, 09E02, 10D01, 10D02, 10D04, 11E01F, 12D01, 12D02E, 17A01G, 17A02G, 17B00G, 17B01G, 17D02, 17D02P, 17D03, 17D03G, 17D04E, 17D04G, 17D04P, 17D06, 17D06P, 29B01, 29D02l, 29D02m, 29D02n, 29D03V, 29D05, 29D05V, 29D06, 29D06V, 29D07, 29D07V, 29D08, 29D08V, 30A01, 30B01,30B02, 30D03, 31D01, 35D03A
**>10% EEAST jobs. No HEMS thresholds = 29 incidents/24hr**<br><br>**18 codes**<br><br>07C03, 09D01, 09E01, 12D01, 17D02, 17D02P, 17D06, 17D06P, 29D02m, 29D02n, 29D05, 29D05V, 29D06, 29D06V, 29D07, 29D07V, 29D08, 29D08V
**>10% EEAST jobs. 60% HEMS patient contact OR 60% HLIDD = 25 incidents/24 hrs**<br><br>16 codes<br><br>07C03, 09D01, 09E01, 12D01, 17D02, 17D02P, 17D06, 17D06P, 29D02n, 29D05, 29D05V, 29D06, 29D06V, 29D07, 29D07V, 29D08V
**>10% EEAST jobs. 70% HEMS patient contact OR 70% HLIDD = 17 incidents/24 hrs**<br><br>9 codes<br><br>07C03, 09E01, 12D01, 17D02P, 17D06, 17D06P, 29D06, 29D06V, 29D07V,
**>10% EEAST jobs. 80% HEMS patient contact OR 80% HLIDD** = **0.4 incidents/24 hrs**<br><br>2 codes<br><br>12D01, 29D07V
**>10% EEAST jobs. 90% HEMS patient contact OR 90% HLIDD** = **0 incidents/24 hrs**<br><br>**0 codes**
