# Supplementary material for: Nine golden codes: improving the accuracy of Helicopter Emergency Medical Services (HEMS) dispatch—a retrospective, multi-organisational study in the East of England
Source: Scand J Trauma Resusc Emerg Med. 2023 Jun 12;31:27. doi: 10.1186/s13049-023-01094-w (PMC10258975; doi:10.1186/s13049-023-01094-w)
Supplement: Supplementary file 3 — Additional file 3. AMPDS codes with significantly high rates of patient contact and/or HLIDD. A table listing the p value for both HLIDD and patient contact for specific codes. [file 13049_2023_1094_MOESM3_ESM.pdf]

Additional File 3: AMPDS codes with significantly high rates of patient contact and/or HLIDD

Code	Description	HEMS taskings 2016-2019					EEAST incidents 2021		
		**N**	**Patient seen rate**		**HLIDD rate**				
			%	P-value	%	P-value	N	HEMS taskings as % of EEAST incidents	Incidents per 24/h
06D02	Breathing problems – Difficulty speaking between breaths	61	73.8%	0.01	-	-	12,584	0.1%	34
**07C03**	**Burns exceeding 18% body area**	**96**	**71.9%**	**0.005**	**-**	**-**	**213**	**11.3%**	**0.6**
09D01	Respiratory Arrest- Ineffective breathing	942	64.3%	<0.001	67.0%	<0.001	1,750	13.5%	5
**09E01**	**Cardiac / Respiratory Arrest - Not Breathing at all**	**2940**	**61.1%**	**<0.001**	**70.0%**	**<0.001**	**5,374**	**13.7%**	**15**
09E02	Cardiac / Respiratory Arrest - Breathing Uncertain (Agonal)	842	63.1%	<0.001	63.3%	<0.001	2,122	9.9%	6
10D01	Not alert with Chest Pains	104	66.4%	0.04	-	-	7,415	0.4%	20
10D02	Chest pain/discomfort - Difficulty speaking between breaths	78	92.3%	<0.001	-	-	9,787	0.2%	27
10D04	Clammy with Chest Pains	90	86.7%	<0.001	-	-	16,443	0.1%	45
11E01F	Choking - Complete obstruction/Ineffective breathing	83	66.3%	<0.001	-	-	237	8.8%	0.6
**12D01**	**Fitting and not breathing**	**91**	**83.5%**	**<0.001**	**89.5%**	**<0.001**	**117**	**19.4%**	**0.3**
12D02E	Convulsions / Fitting - CONTINUOUS or MULTIPLE fitting - Epileptic or Previous diagnosis of fitting	275	65.1%	0.013	-	-	5,552	1.2%	15
17A01G	Fallen - Marked (*) NOT DANGEROUS PROXIMAL or DISTAL body area with deformity still on ground	76	84.2%	<0.001	85.9%	<0.001	1,260	1.5%	4
17A02G	Fallen - Not Dangerous Proximal Injuries still on ground	55	80.0%	0.001	-	-	8,154	0.2%	22
17B00G	Falls - Override - On the ground or floor	66	81.8%	<0.001	75.9%	0.006	n/a (a)		
17B01G	Fall - possibly dangerous area injuries - still on ground	97	76.3%	0.003	-	-	14,253	0.2%	39
17D02	Falls - Arrest	227	-	-	67.8%	0.01	487	11.7%	1
**17D02P**	**Falls - Arrest - Public place (street, parking garage, market)**	**50**	**74.0%**	**0.021**	**-**	**-**	**63**	**19.8%**	**0.2**

17D03	Unconscious post fall	282	64.5%	0.019	-	-	745	9.5%	2
17D03G	Falls - Unconscious - On the ground or floor	77	72.7%	0.008	-	-	n/a (a)		
17D04E	Falls - Not alert - Environmental problems (rain, heat, cold)	73	78.1%	<0.001	-	-	1,313	1.4%	4
17D04G	Falls - Not alert - On the ground or floor	283	70.7%	<0.001	-	-	15,090	0.5%	41
17D04P	Falls - Not alert - Public place (street, parking garage, market)	184	73.9%	<0.001	-	-	3,156	1.5%	9
**17D06**	**Long fall**	**75**	**73.3%**	**0.007**	**-**	**-**	**168**	**11.2%**	**0.5**
**17D06P**	**Long fall - Public place (street, parking garage, market)**	**61**	**73.8%**	**0.012**	**-**	**-**	**58**	**26.3%**	**0.2**
29B01	Injuries	137	70.8%	0.002	-	-	773	4.4%	2
29D02l	Traffic / Transportation Incidents - HIGH MECHANISM (k through t) - Vehicle Vs Bicycle/ Vehicle Vs Motorcycle	517	72.0%	<0.001	72.3%	<0.001	1,462	8.8%	4
29D02m	Traffic / Transportation Incidents - HIGH MECHANISM (k through t) - Vehicle Vs Pedestrian	554	60.7%	<0.001	-	-	1,059	13.1%	3
29D02n	Traffic / Transportation Incidents - HIGH MECHANISM (k through t) - Ejection	112	63.4%	<0.001	-	-	68	41.2%	0.2
29D03V	Traffic / Transportation Incidents - HIGH VELOCITY impact - Multiple patients	131	68.7%	0.011	-	-	519	6.3%	1
29D05	Trapped victim	455	66.37 %	<0.001	65.2%	0.003	258	44.1%	0.7
29D05V	Traffic / Transportation Incidents - Trapped victim - Multiple patients	222	66.37 %	<0.001	-	-	106	52.4%	0.3
**29D06**	**Arrest post RTC**	**320**	**64.1%**	**0.021**	**75.6%**	**<0.001**	**176**	**45.5%**	**0.5**
**29D06V**	**Traffic / Transportation Incidents - Arrest - Multiple patients**	**67**	**71.7%**	**0.022**	**77.1%**	**0.004**	**28**	**59.8%**	**0.1**
29D07	Unconscious post RTC	406	65.2%		65.1%	0.009	188	54.0%	0.5
**29D07V**	**Traffic / Transportation Incidents - Unconscious - Multiple patients**	**119**	**76.5%**	**<0.001**	**80.2%**	**<0.001**	**28**	**106% (b)**	**0.1**
29D08	Not alert with noisy breathing (abnormal)	243	63.5%	<0.001	-	-	204	29.8%	0.6
29D08V	Traffic / Transportation Incidents - Not alert with noisy breathing (abnormal) - Multiple patients	93	69.9%	0.018	-	-	31	75.0%	0.1

30A01	Marked (*) NOT DANGEROUS PROXIMAL or DISTAL body area with deformity	142	78.9%	<0.001	76.8%	<0.001	1,369	2.6%	4
30B01	POSSIBLY DANGEROUS body area	187	78.1%	<0.001	-	-	3,499	1.3%	10
30B02	SERIOUS haemorrhage	80	63.8%	<0.001	-	-	1,167	1.7%	3
30D03	Not alert with traumatic injuries	330	72.4%	<0.001	-	-	5,370	1.5%	15
31D01	Unconscious – AGONAL/INEFFECTIVE BREATHING	147	66.0%	0.041	76.3%	<0.001	609	6.0%	2
35D03A	HCP and AED on scene (immediate life threat)	170	77.7%	<0.001	-	-	n/a (a)		

Nine ‘golden codes’ highlighted **bold**

Notes: (a) Code not present in 2021 EEAST data. (b) These are estimated percentages using data from different time periods, so >100% can occur. Also, the count of HEMS taskings can exceed EEAST incidents if more than one HEMS crew is tasked to the same incident.
